# Exploration of sensing data to realize intended odor impression using mass spectrum of odor mixture

**DOI:** 10.1371/journal.pone.0273011

**Published:** 2022-08-17

**Authors:** Daisuke Hasebe, Manuel Alexandre, Takamichi Nakamoto

**Affiliations:** 1 School of Engineering, Tokyo Institute of Technology, Yokohama, Kanagawa, Japan; 2 Institute of Innovation Research, Tokyo Institute of Technology, Yokohama, Kanagawa, Japan; University of Bonab, ISLAMIC REPUBLIC OF IRAN

## Abstract

Recently, olfactory information on odorants has been associated with their corresponding molecular features. Such information has been obtained by predicting the sensory test evaluation scores from the molecular structure parameters or the sensing data. On the other hand, we develop a method of the prediction of molecular features corresponding to the odor impression. We utilize a machine-learning-based odor predictive model introduced in our previous research, and we propose a mathematical model for exploring the sensing data space. By using mass spectrum as sensing data in the predictive model, we can represent predicted mass spectrum as those of an odor mixture, and the mixing ratio can be obtained. We show that the mass spectrum of apple flavor with enhanced ‘fruit’ and ‘sweet’ impressions can be obtained using 59 and 60 molecules respectively by using our analysis method.

## Introduction

The sense of smell is important partly close to the instincts of animal a species, to detect dangers such as natural enemies and poisonous substances. Humans perceive hundreds of thousands of odorants through about 400 types of olfactory receptors expressed in olfactory nerve cells. Only a single type of sensory receptor is expressed on an olfactory nerve cell, and the response patterns of the same type of olfactory receptor to an odorant are the same and have the same odor selectivity [1]. Then, that the brain processes the response patterns of olfactory nerve cells that are differently excited by odorants having various types of physicochemical information.

Conventionally, a human sensory evaluation tests has been performed to determine how an odor impression is perceived. Because sensory tests require much time and labor, attempts have been made to predict sensory test data from physicochemical parameters or sensing data. As an early attempt, Khan et al. performed dimensionality reduction with principal component analysis for both physicochemical structural information and odor perception data, and compared their principal components [2]. As a result, it was found that the first principal component of physicochemical parameters and the first principal component of perception correlate the most, and it was concluded that the first principal component for perceptual data was pleasantness. Guo et al. proposed a convolutional LSTM (Long Short Term Memory) that predicts sensory test data using sensing data obtained from an electronic nose (E-nose) [3]. E-nose is composed of multiple sensors. For the prediction, the combination of sensors should be able to capture odor perception.

Many studies use physicochemical parameters to predict odor perception [4, 5], but they are not applicable to odor mixtures. We used the mass spectrum to predict odor impression [6, 7] because the mass spectrum of the mixed odor can be obtained by performing a linear combination of component mass spectra. We improved its prediction accuracy by increasing the number of data used for pretraining a part of the model [8]. The prediction accuracy reached 0.90 in terms of correlation coefficient.

The next problem to be solved is the prediction of sensing data from odor impression. If the sensing data can be predicted from the basis of a given odor impression, it is possible to generate the desired scent automatically based on sensing data. We have already proposed a method to explore the sensing feature space using an odor impression predictive model [9]. The sensing data used here is again the mass spectrum. However, such data is limited to exploration of mass spectrum feature space and the approximation accuracy using odorant molecules has not been investigated. In this paper, we extended our conference paper in Ref. [9] in terms of mass spectrum approximation using odorant molecules as well as detailed exploration algorithms.

## Data

A mass spectrum is obtained by mass spectrometry, which is used for the structural analysis of molecules. In mass spectrometry, a sample is ionized, ions are separated according to their mass-to-charge ratio (m/z), and then a pattern of intensities with respect to m/z that is unique to a molecule is obtained under the same condition. From the principle of mass spectrometry, a linear superposition using the mixing ratio holds. We used the mass spectra of 2106 odorant molecules from the NIST Chemistry WebBook database as physicochemical data to predict sensory data [10]. The low m/z region with peaks for the solvent molecule and the high-m/z region with little contribution to odor perception were removed, then 51–262 m/z region of the mass spectra was used.

The sensory test is a method of evaluating the quality of an object by the five human senses. In this study, we used odor impression scores for 21 odor descriptors from a sensory test dataset for olfactory information called the DREAM dataset [11]. The descriptors are ‘pleasantness’, ‘intensity’, ‘bakery’, ‘sweet’, ‘fruit’, ‘fish’, ‘garlic’, ‘spices’, ‘cold’, ‘sour’, ‘burnt’, ‘acid’, ‘warm’, ‘musky’, ‘sweaty’, ‘ammonia/urinous’, ‘decayed’, ‘wood’, ‘grass’, ‘flower’ and ‘chemical’. Fifty-five subjects evaluated their odor impressions of up to 21 of these descriptors. The score of the descriptor that a subject did not evaluate was regarded as 0. To associate the mass spectrum of a molecule with the sensory test data, the sensory test data was averaged across the subjects. We used the data of 383 molecules since both their mass spectra and the sensory test data are available.

## Method to predict mass spectrum from odor impression

We have successfully mapped the mass spectrum onto sensory data as mentioned in **Introduction**. However, different mass spectra sometimes converge to similar points in the sensory data space. Thus, it is necessary to solve the inverse problem.

**Fig 1:**
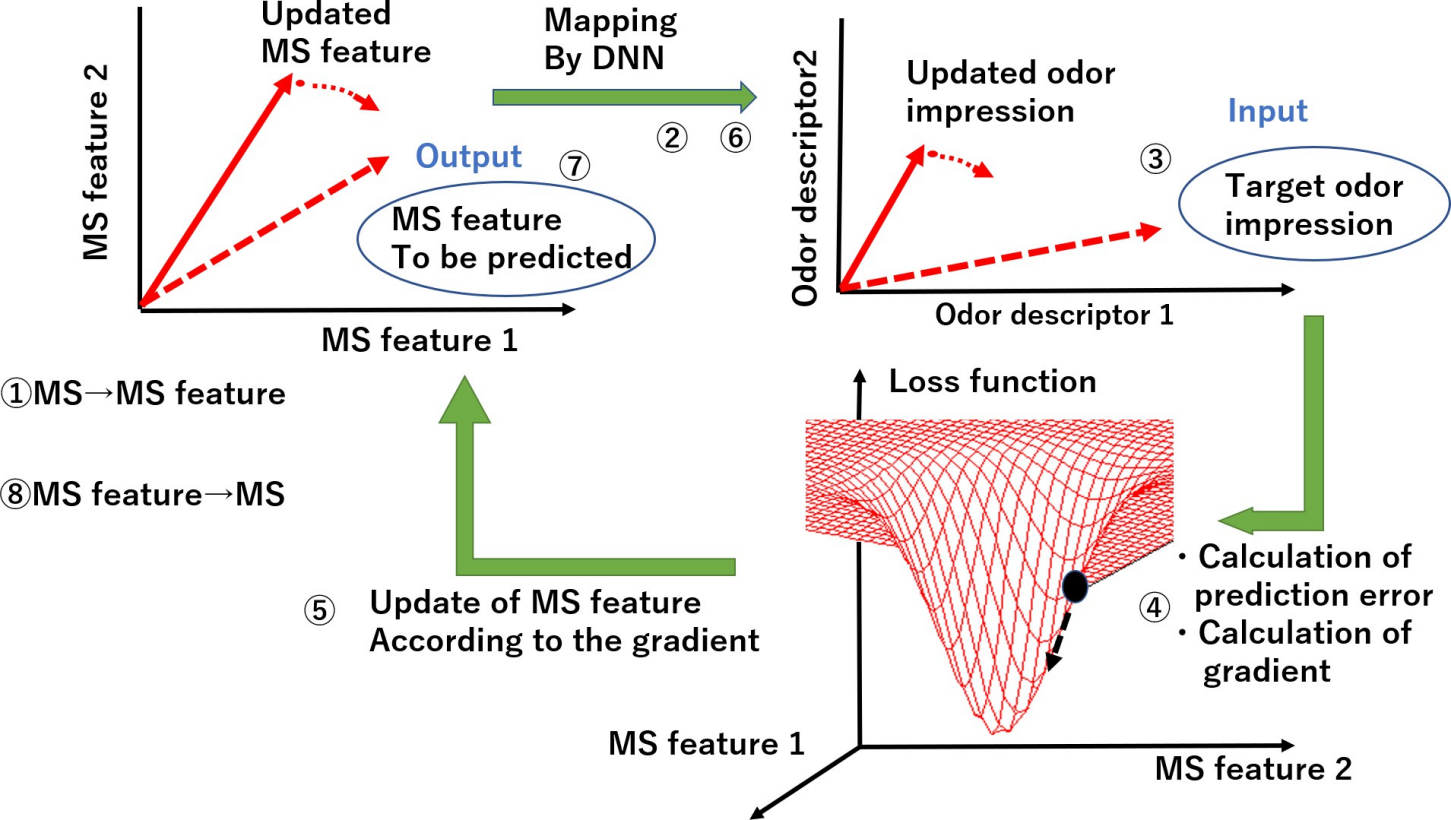
Principle of the prediction from odor impression to mass spectrum.

Overall algorithm is illustrated in **Fig 1**. The mass spectrum is initially set as its feature vector (MS feature) at first and then it is repeatedly updated to map the point in sensory data space onto MS feature space. The sensory data after convergence should match specified ones. The procedure is as follows.

1. Calculate the MS feature with a trained MS autoencoder.
2. Calculate odor impression using deep neural network (DNN)
3. Determine the target odor impression [input].
4. Calculate the gradient in the MS feature space on the basis of loss function, i.e., the error between the target and the predicted odor impression.
5. Update the MS feature according to the gradient.
6. Calculate odor impression corresponding to the new MS feature using DNN.
7. After iteration (4–6), choose a MS feature that has minimal error in step 4 [output].
8. The original mass spectrum is recovered from the MS feature.

The cost function and the gradient derivation are explained in the next section.

## Odor predictive model

**Fig 2:**
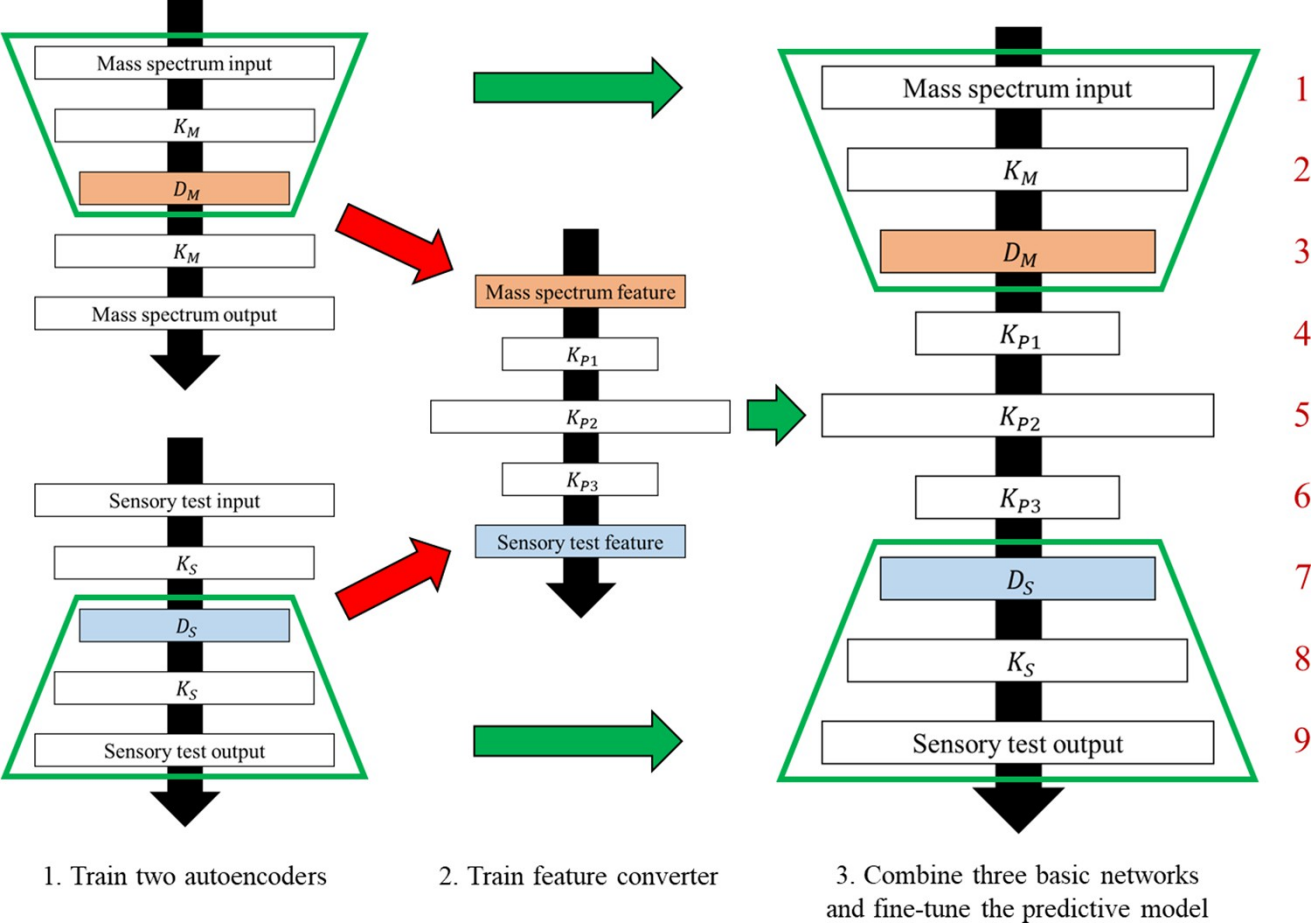
Schematic diagram of the odor impression predictive model.

The odor predictive model is a neural network that predicts odor impression scores of a molecule from the mass spectrum. It is composed of three basic networks: two deep autoencoders and a multi-layer perceptron as shown in **Fig 2**. We used five-layer autoencoders with a non-linear sigmoid activation function, to reduce the dimensions of both mass spectrum data and sensory test data. The dimensionally reduced features were mapped by a five-layer perceptron, also with a sigmoid activation function. Finally, a nine-layer odor impression predictive model was generated by combining the encoder part of the autoencoder for mass spectrum data, the feature-to-feature mapping, and the decoder part of the autoencoder for sensory test data.

Here, we mathematically deal with the odor predictive model. Since the three basic neural networks are non-linear multi-layer perceptrons with the same activation function, the odor impression predictive model can also be considered as a multi-layer perceptron. Let the vector ***X***^(*k*)^ of the *k*th layer and the activation ***A***^(*k*)^ in the layer be expressed as follows:

$$X^{\left(k\right)}=(x_{1}^{k},x_{2}^{k},\cdots ,x_{n(k)}^{k}),$$ (1)

$$A^{\left(k\right)}=(a_{1}^{k},a_{2}^{k},\cdots ,a_{n(k+1)}^{k})=W^{\left(k,k+1\right)}X^{\left(k\right)}+B^{\left(k,k+1\right)}.$$ (2)

where *n*(*k*) is the number of nodes, *W*^(*k*,*k*+1)^ is the weight matrix and ***B***^(*k*,*k*+1)^ is the bias vector in the *k*th layer. Using an element-wise sigmoid function

$$Z^{\left(k,k+1\right)}(A^{\left(k\right)})=\left(\zeta_{1}^{\left(k,k+1\right)}(a_{1}^{k}),\zeta_{2}^{\left(k,k+1\right)}(a_{2}^{k}),\cdots ,\zeta_{n(k+1)}^{\left(k,k+1\right)}(a_{n(k+1)}^{k})\right),$$ (3)

We present the (*k*+1)th layer as

$$X^{\left(k+1\right)}=Z^{\left(k,k+1\right)}(A^{\left(k\right)})=Z^{\left(k,k+1\right)}(W^{\left(k,k+1\right)}X^{\left(k\right)}+B^{\left(k,k+1\right)})\equiv f^{\left(k,k+1\right)}(X^{\left(k\right)}),$$ (4)

where ***f***^(*i*,*j*)^(***X***^(*i*)^) is a function that maps the *i*th layer onto the *j*th layer. This allows ***X***^(9)^ to be

$$X^{\left(9\right)}=(f^{\left(8,9\right)}\circ f^{\left(7,8\right)}\circ \cdots \circ f^{\left(1,2\right)})(X^{\left(1\right)})=f^{\left(1,9\right)}(X^{\left(1\right)})$$ (5)

as a function for the input ***X***^(1)^.

The model training process consists of pretraining of three basic networks (two autoencoders and a feature converter) and fine-tuning after combining them. The 5-layer autoencoder for mass spectrum data was trained and evaluated by five-fold cross validation using 500,000 synthetic mass spectra, each of which was linearly combined at random ratios using three randomly chosen mass spectra. Then, the features of the 383 mass spectra that are common in both datasets were obtained through the trained autoencoder. The 5-layer autoencoder for sensory test data was trained using the 383 molecules and it was evaluated by 12-fold cross validation. Similarly, the feature converter was trained and tested by 12-fold cross validation with 383 dimensionally reduced feature pairs. Finally, the model was fine-tuned after pre-training and combining the three basic networks. The entire training process updated the weights and biases 2000 times by the backpropagation method to minimize the sum of squares error using the stochastic gradient descent method. In the update formula, the inertial term to suppress sudden changes and the regularization term to avoid saturation and overfitting were adopted. The L2 norm (Ridge regression) was used for the regularization term [12].

The entire data was normalized. Each mass spectrum was normalized by dividing all spectra by the maximum value because the mass spectrum has a unique pattern for each molecule under the same condition. The entire sensory data was normalized by dividing all scores by the total maximum value. These normalizations were carried out because we adopted sigmoid activation. These normalizations ensure that the maximum value of the data does not exceed 1. Weights and biases were initialized using Xavier’s initialization [13].

The hyper parameters of the odor prediction model are listed in **Table 1**. The subscripts *M*, *S*, *P* and *F* of the hyperparameters indicate that they are those of the autoencoder for mass spectrum data, the autoencoder for sensory data, the feature converter, and the fine-tuning, respectively. The numbers of nodes in the first hidden layer of the autoencoders *K*_*M*_ and *K*_*S*_; the numbers of nodes in the second hidden layer of the autoencoders *D*_*M*_ and *D*_*S*_; the numbers of nodes in the first to third hidden layers of the feature converter *K*_*P*1_, *K*_*P*2_ and *K*_*P*3_; the coefficients of the regularization terms *λ*_*M*_, *λ*_*S*_, *λ*_*P*_ and *λ*_*F*_; the learning rates in the back propagation *η*_*M*,*τ*_, *η*_*S*,*τ*_, *η*_*P*,*τ*_ and *η*_*F*,*τ*_; the coefficients of the inertia terms *α*_*M*_, *α*_*S*_, *α*_*P*_ and *α*_*F*_ are included in the table. *τ* is the number of iterations in each case.

**Table 1:** Hyperparameters of the odor impression predictive model.

Hyperparameter	Range of search	Step size	The value used
*K*_*M*_	20−150	10	85
*D*_*M*_	5−90	5	70
*λ*_*M*_	7.5−7.5×10^−10^	×10^−1^	7.5×10^−5^
*η*_*M*,*τ*_	−	−	0.5×0.99^*τ*^
*α*_*M*_	−	−	0.3
*K*_*S*_	5−25	5	20
*D*_*S*_	5−25	5	15
*λ*_*S*_	0.2−2.0×10^−9^	×10^−1^	2.0×10^−9^
*η*_*S*,*τ*_	−	−	0.4×0.99^*τ*^
*α*_*S*_	−	−	0.3
*K*_*P*1_	10−100	5	30
*K*_*P*2_	10−100	5	70
*K*_*P*3_	10−100	5	45
*λ*_*P*_	1.0−1.0×10^−8^	×10^−1^	1.0×10^−5^
*η*_*P*,*τ*_	−	−	0.4×0.99^*τ*^
*α*_*P*_	−	−	0.25
*λ*_*F*_	−	−	2.0×10^−5^
*η*_*F*,*τ*_	−	−	0.35×0.99
*α*_*F*_	−	−	0.025

## Mass spectrum that realizes intended odor impression

Owing to the large number of dimensions of the mass spectrum, the complexity of odor perception, and the instability of sensory test evaluation, it is difficult to construct a model that directly predicts the mass spectrum from the odor impression. We examined an approach to predicting the input space of the odor impression predictive model by the gradient descent method. However, since most of the intensities of a mass spectrum are 0 and other peaks have positive values, it has been found from a preliminary experiment that it is difficult to search the mass spectrum directly using the gradient descent method. Therefore, we focused on a reduced MS feature space instead of raw mass spectrum space. The mass spectrum feature space is the middle layer of the autoencoder for mass spectrum data.

The method of exploring the mass spectrum feature space using the gradient descent method is described below. The odor impression corresponding to the value in the mass spectrum feature space ***X***^(3)^ is expressed as the following equation:

$$X^{\left(9\right)}=(f^{\left(8,9\right)}\circ f^{\left(7,8\right)}\circ \cdots \circ f^{\left(3,4\right)})(X^{\left(3\right)})=f^{\left(3,9\right)}(X^{\left(3\right)}).$$ (6)

The derivative of ***f***^(3,9)^ with respect to ***X***^(3)^ is

$$\frac{df^{\left(3,9\right)}}{dX^{\left(3\right)}}=\frac{df^{\left(8,9\right)}}{dX^{\left(8\right)}}\frac{dX^{\left(8\right)}}{dX^{\left(7\right)}}\cdots \frac{dX^{\left(4\right)}}{dX^{\left(3\right)}}=\frac{df^{\left(8,9\right)}}{dX^{\left(8\right)}}\frac{df^{\left(7,8\right)}}{dX^{\left(7\right)}}\cdots \frac{df^{\left(3,4\right)}}{dX^{\left(3\right)}},$$ (7)

$$\frac{df^{\left(k,k+1\right)}}{dX^{\left(k\right)}}=\frac{dZ^{\left(k,k+1\right)}}{dA^{\left(k\right)}}\frac{dA^{\left(k\right)}}{dX^{\left(k\right)}}=\left(\begin{array}{ccc} \frac{d\zeta_{1}^{\left(k,k+1\right)}}{da_{1}^{k}} & & 0\\ & \ddots & \\ 0 & & \frac{d\zeta_{n(k+1)}^{\left(k,k+1\right)}}{da_{n(k+1)}^{k}} \end{array}\right)W^{\left(k,k+1\right)},$$ (8)

$$\frac{d\zeta_{i}^{\left(k,k+1\right)}}{da_{i}^{k}}=\zeta_{i}^{\left(k,k+1\right)}(a_{i}^{k})\left\{1-\zeta_{i}^{\left(k,k+1\right)}(a_{i}^{k})\right\}.$$ (9)

In the gradient descent updates, the search space ***X***^(3)^ is replaced with

$$X^{\left(3\right)}-\eta_{G,\tau }\frac{dL(X^{\left(9\right)})}{dX^{\left(3\right)}}=X^{\left(3\right)}-\eta_{G,\tau }\frac{dL(X^{\left(9\right)})}{dX^{\left(9\right)}}\frac{dX^{\left(9\right)}}{dX^{\left(3\right)}}=X^{\left(3\right)}-\eta_{G,\tau }\frac{dL(X^{\left(9\right)})}{dX^{\left(9\right)}}\frac{df^{\left(3,9\right)}}{dX^{\left(3\right)}},$$ (10)

where

$$\eta_{G,\tau }=\eta_{0}\times \gamma^{\tau },$$ (11)

and

$$L(X^{\left(9\right)})=\frac{1}{2}\sum_{i=1}^{n(9)}m_{i}{\left(t_{i}-x_{i}^{9}\right)}^{2},$$ (12)

where *L*(***X***^(9)^) is the weighted sum of squares error (WSE), *η*_*G*,*τ*_ is the update rate, *η*_0_ is the initial value, *γ* is the attenuation rate, and *m*_*i*_ is a weight for the *i*th descriptor’s score. By repeating the update (10), we obtain the optimal ***X***^(3)^.

The mass spectrum that realizes the searched mass spectrum feature was determined by a line search that incorporated randomness. The algorithm is **Algorithm 1** found in the supplement section (**S1 Fig**). It is necessary to maintain good accuracy to restore the mass spectrum from its feature. **Algorithm 1 in S1 Fig** has a higher accuracy than the decoder part of autoencoder even if it consumes a longer calculation time.

The mass spectrum explored using the **Algorithm 1 in S1 Fig** can be considered as a synthetic mass spectrum. Therefore, since the synthetic mass spectrum can be represented by a linear combination of the component mass spectra, we obtained the mixture composition by the nonnegative constrained least squares regression method.

## Results

### Training of the odor impression predictive model

We trained the odor impression predictive model by the method described above. To perform the feature search experiment by the gradient descent method, it is necessary to select one of the 12 cross-validation divisions. The maximum value of the correlation coefficient is 0.93, which is higher than those in previous reports (0.76 in [6], 0.86 and 0.90 in [8]). This is due to both the increase in the amount of training data of the autoencoder for mass spectrum and the increase in the number of molecules included in the sensory test data compared with previous reports [6, 8].

**Fig 3:**
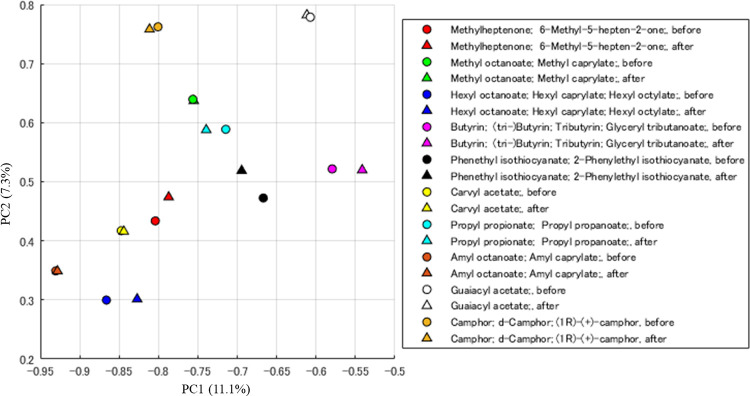
PCA diagram of MS features before and after applying the gradient descent algorithm to the 10 molecules in the DREAM dataset. The circle are mass spectral features obtained by reducing the dimension of the original mass spectra. The triangles are mass spectral features searched by applying the gradient descent method, predicted from odor impression. All plots are obtained using the first and second principal components with proportion of variance.

We applied the gradient descent algorithm to the 10 molecules contained in the DREAM dataset to predict the mass spectrum from the target odor impression. The parameters of the search were *η*_*G*,*τ*_ = 0.1×0.99^*τ*^ and *m*_*i*_ = 1 for all *i*. The gradient descent was repeated 2000 times. The result is shown in **Fig 3**. The prediction accuracy of the model is not perfect, so it contains some prediction errors in the odor descriptor space. The mass spectrum feature search algorithm by the gradient descent method can work to search for a feature that minimize this prediction error. However, **Fig 3** shows that the gradient descent method has little effect on changing the location of the corresponding mass spectrum feature, which means that the trained odor impression predictive model has sufficiently high accuracy.

### Model performance for odor mixture

The model above was trained basically using single molecules, so it is necessary to verify whether the odor impression is predicted correctly with respect to the mass spectrum. Therefore, we evaluated the performance of the model by synthesizing two single molecules at continuously varying ratios. We adopted citral and vanillin as the two molecules included in the current dataset. The former is the scent of lemon and the latter is the scent of vanilla.

**Fig 4:**
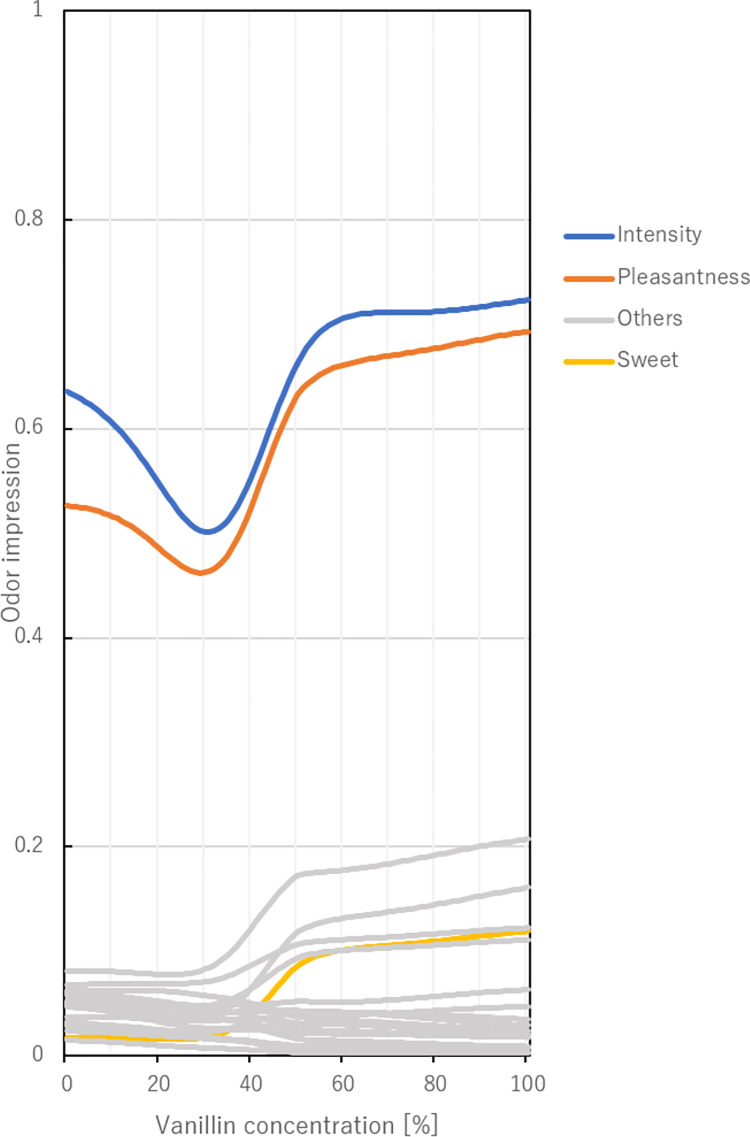
Changes in the scores of the odor descriptors for each mixing ratio of vanillin and citral. The horizontal axis shows the mixing ratio of vanillin.

**Fig 4** shows the result of the change in odor impression across the range of odor mixing ratios. The odor impression change was calculated using **Eq (6)**, where the mass spectrum feature of citral-vanillin mixture was put into ***X***^(3)^. The mass spectra used here were normalized ones since they come from the NIST database. The odor impression was predicted for every 1% change in the mixing ratio. The output of the model showed smooth changes in odor impression. The most rapid changes were observed when the mixing ratio was approximately 50%, whereas a gradual change was confirmed near the end points. Regarding the descriptors ‘intensity’ and ‘pleasantness’, the impression scores tended to decrease at the concentration points of 0 to 30%, and tended to increase monotonically in the other parts. From this, it is considered that a molecule is much more dominant in the odor impression when its concentration is close to 100%. It is expected that the trained model can express the impression score using the pattern of mass spectrum of the mixture, but this finding should be verified by a sensory test.

### Application to a complex odor mixture

**Fig 5:**
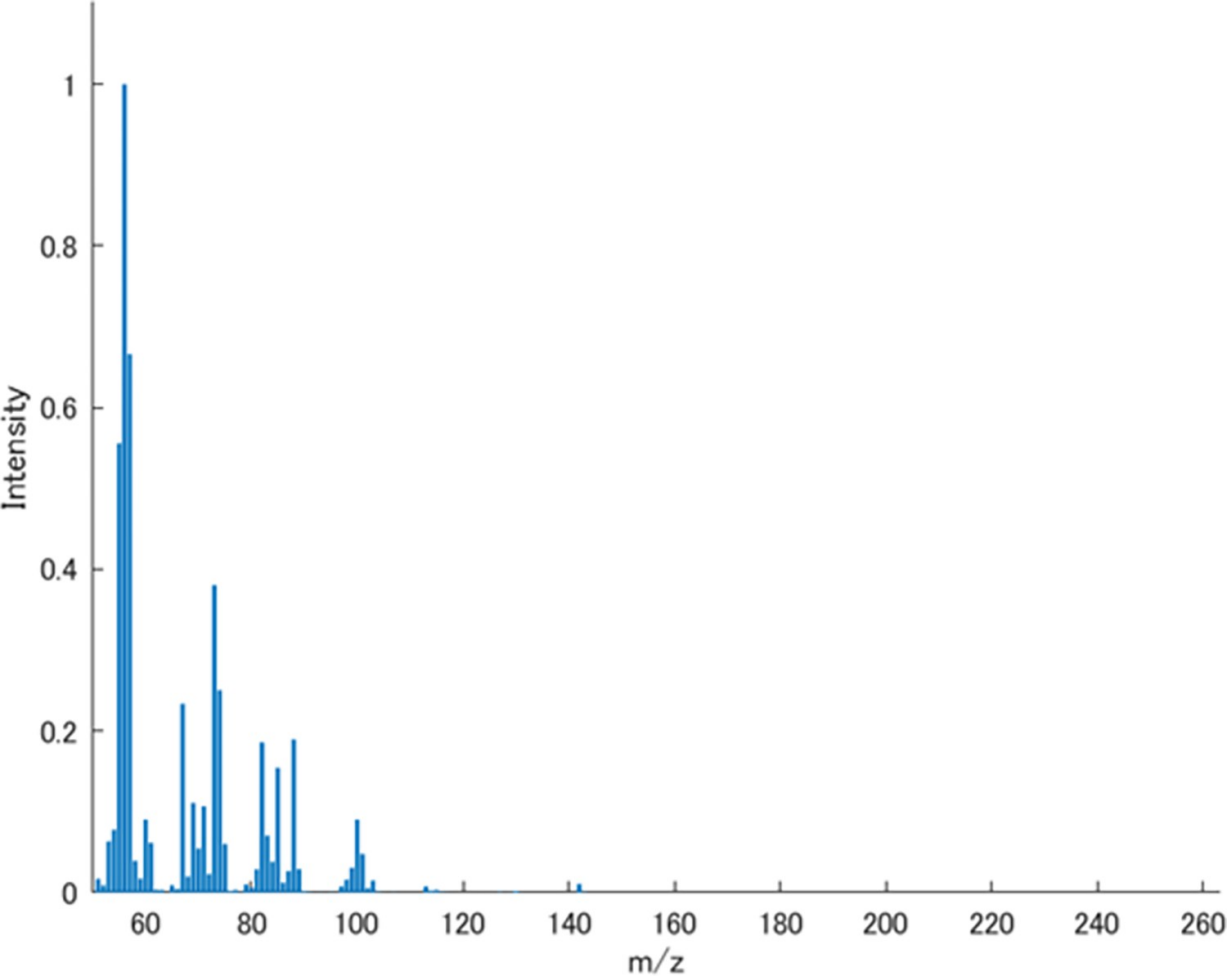
Mass spectrum of the virtual apple flavor.

In this study, we applied the input space search methods using the odor impression predictive model described above to an apple flavor, an odor mixture with its recipe, given in Ref. [14]. The mass spectrum of the apple flavor is shown in **Fig 5**. The apple flavor consists of nine odor component molecules, and its mass spectrum was calculated by considering the ratios (v/v) of ingredients as the coefficients of linear combination. The apple flavor is ‘virtual’ since normalized mass spectra of nine ingredients in the database were used to calculate its mass spectrum. The sensory data was obtained using the odor impression predictive model. Before applying the input space search methods, we changed the target odor impression of the apple flavor for the two odor descriptors (‘fruit’ and ‘sweet’).

**Fig 6:**
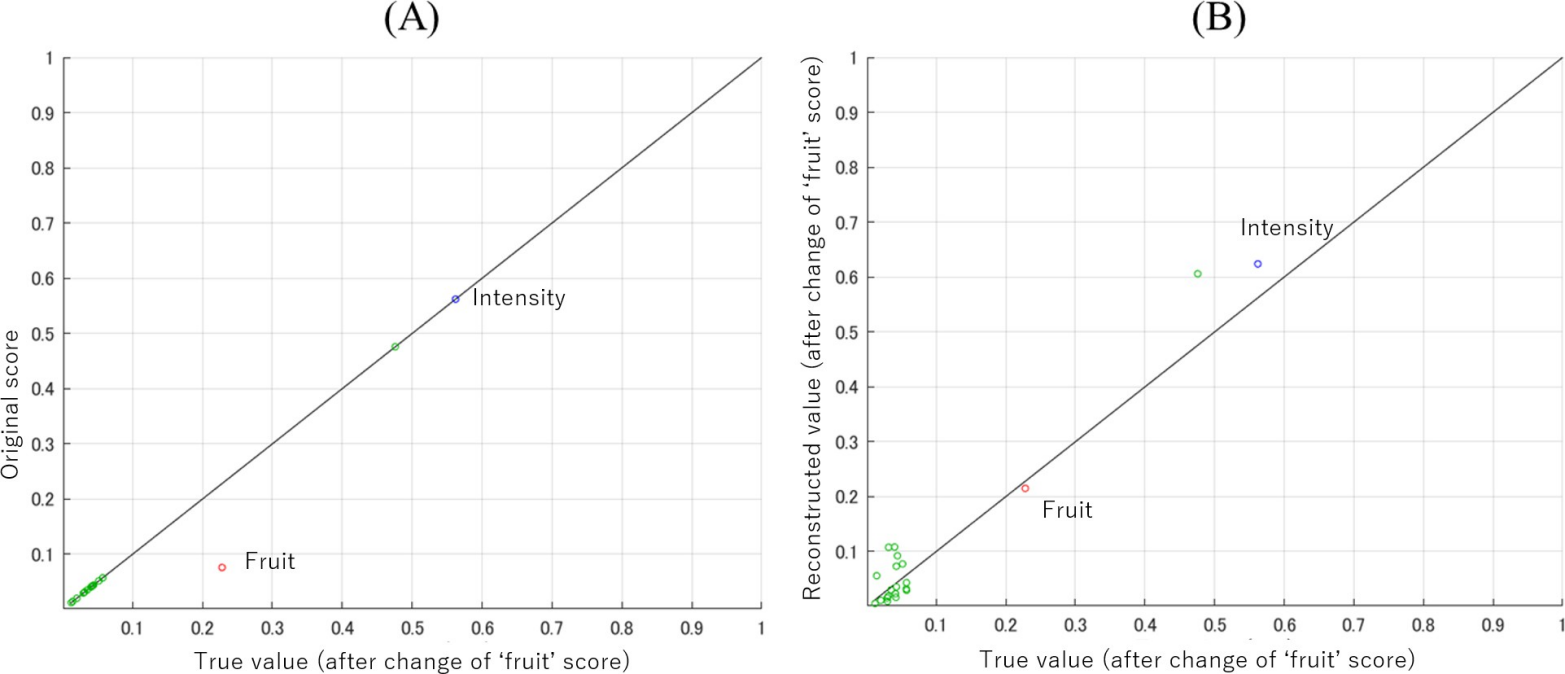
Predicted sensory test score **(A)**: The score for the original apple flavor (vertical axis) and the true value with the score of ‘fruit’ was multiplied by 3.0 (horizontal axis). **(B)**: The score for the explored mass spectral feature (vertical axis) and the true value with the score of ‘fruit’ was multiplied by 3.0 (horizontal axis). The blue, red and green plots are those of ‘intensity’, ‘fruit’ and others, respectively.

**Fig 7:**
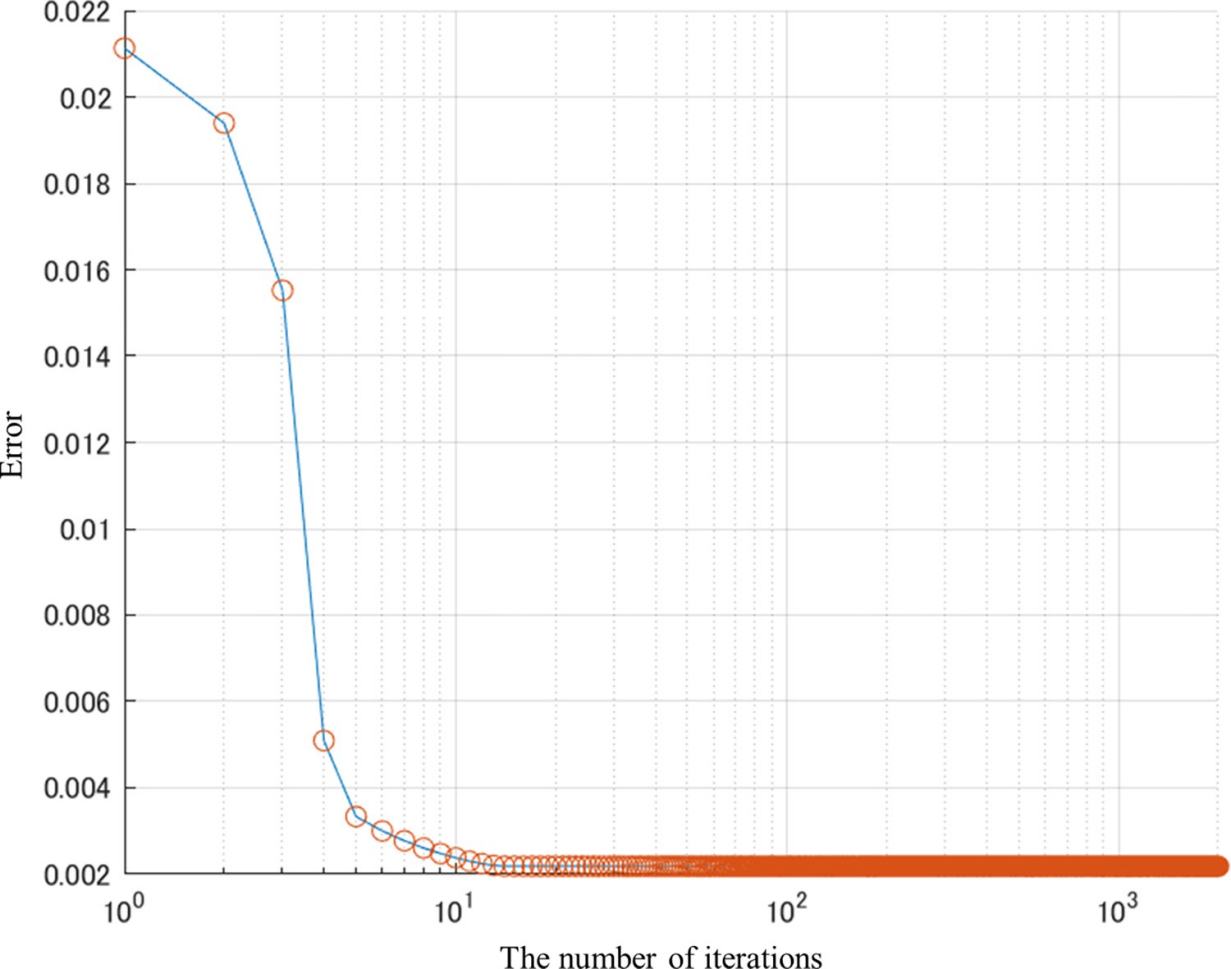
Transition of errors in sensory space in the gradient descent method for ‘fruit’.

First, we conducted an experiment on the odor descriptor ‘fruit’. **Fig 6(A)** shows the paired plots for the predicted sensory data scores and the virtual true values in which the odor descriptor ‘fruit’ has a tripled predicted value as the true value and the others have the same values as the predicted ones. The gradient descent method was applied to obtain the true value. The parameters used here were *η*_*G*,*τ*_ = 0.1×0.99^*τ*^ and *m*_*i*_ = 1 for ‘fruit’ and *m*_*i*_ = 0.05 for the other descriptors. **Fig 6(B)** shows the result in the sensory data space after optimization by the gradient descent method. The specified odor impression for ‘fruit’ was obtained since the red plot in the figure is on the diagonal line. On the other hand, the other descriptors have different odor impressions and deviated from the diagonal line. It seems that the other impressions changed according to the correlation between the descriptors to obtain the true value of ‘fruit’ with a heavier penalty. The error transition in the sensory data space during the process is shown in **Fig 7**. It can be seen that the error converged in about 10 iterations.

**Fig 8:**
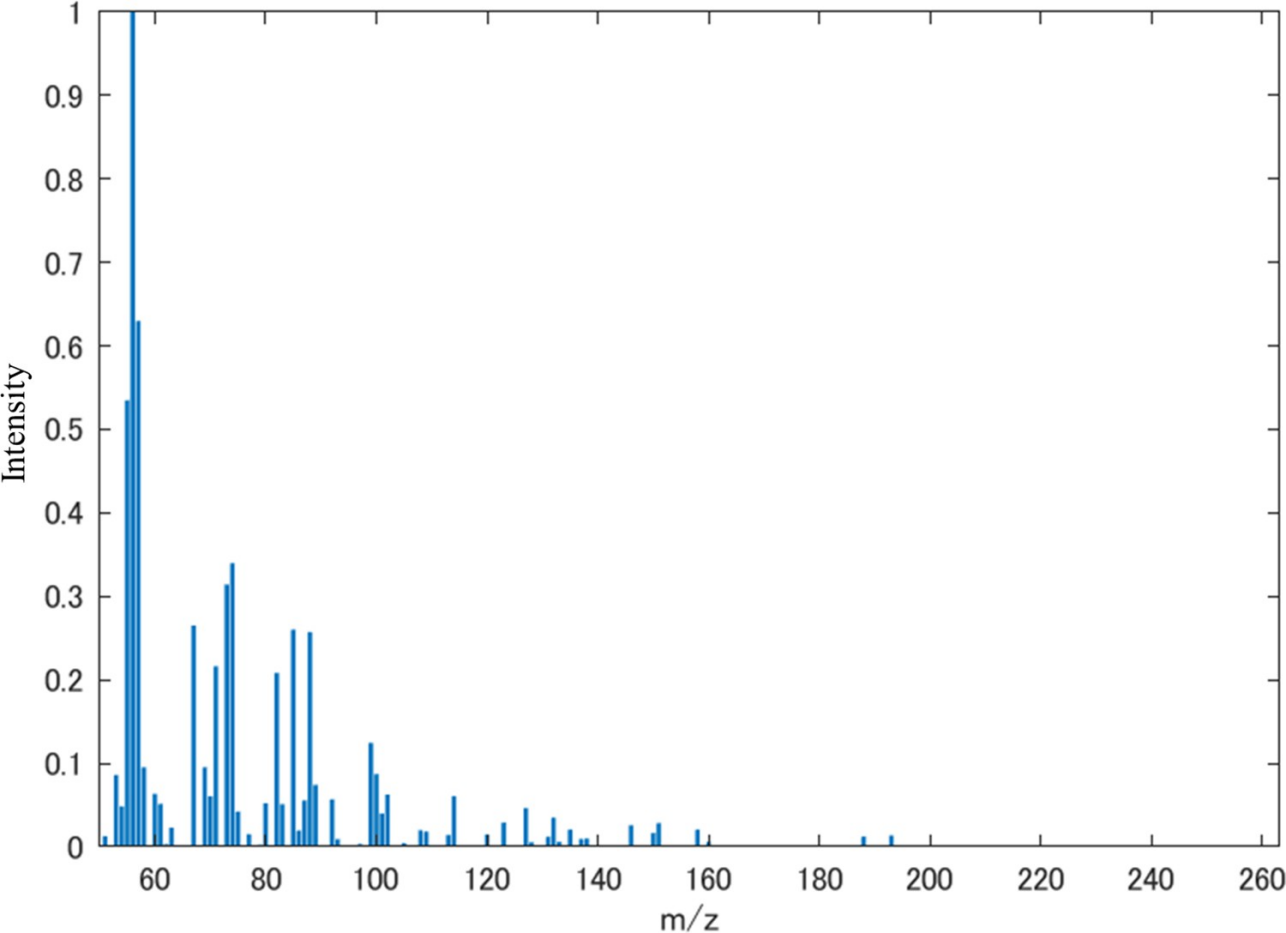
Mass spectrum obtained by applying Algorithm 1 in S1 Fig to the explored mass spectrum feature in the experiment for ‘fruit’.

We searched for a mass spectrum that provides the explored features by applying the **Algorithm 1** (**S1 Fig**). The parameters of the search were Δ = 0.5×0.9999^*τ*^ and the number of iterations was 100,000, where Δ is the update rate described in **Algorithm 1** (**S1 Fig**). The data obtained through **Algorithm 1 in S1 Fig** was close to the exact one since **Algorithm 1 in S1 Fig** is based on the method requiring numerous searched points. The resulting mass spectrum is shown in **Fig 8**. For the mass spectrum, the sum of squares of error on the mass spectrum feature space obtained through the autoencoder was 0.0325, which corresponds to an average error of about 2% in the dimension of each m/z feature because the feature dimension is 70. Such mass spectrum might express mostly correct and less noisy peaks with low intensities. In addition, since the obtained mass spectrum contained peaks of the high-m/z region, it is considered that the apple flavor with an increased ‘fruit’ score should have additional fruity molecules.

**Fig 9:**
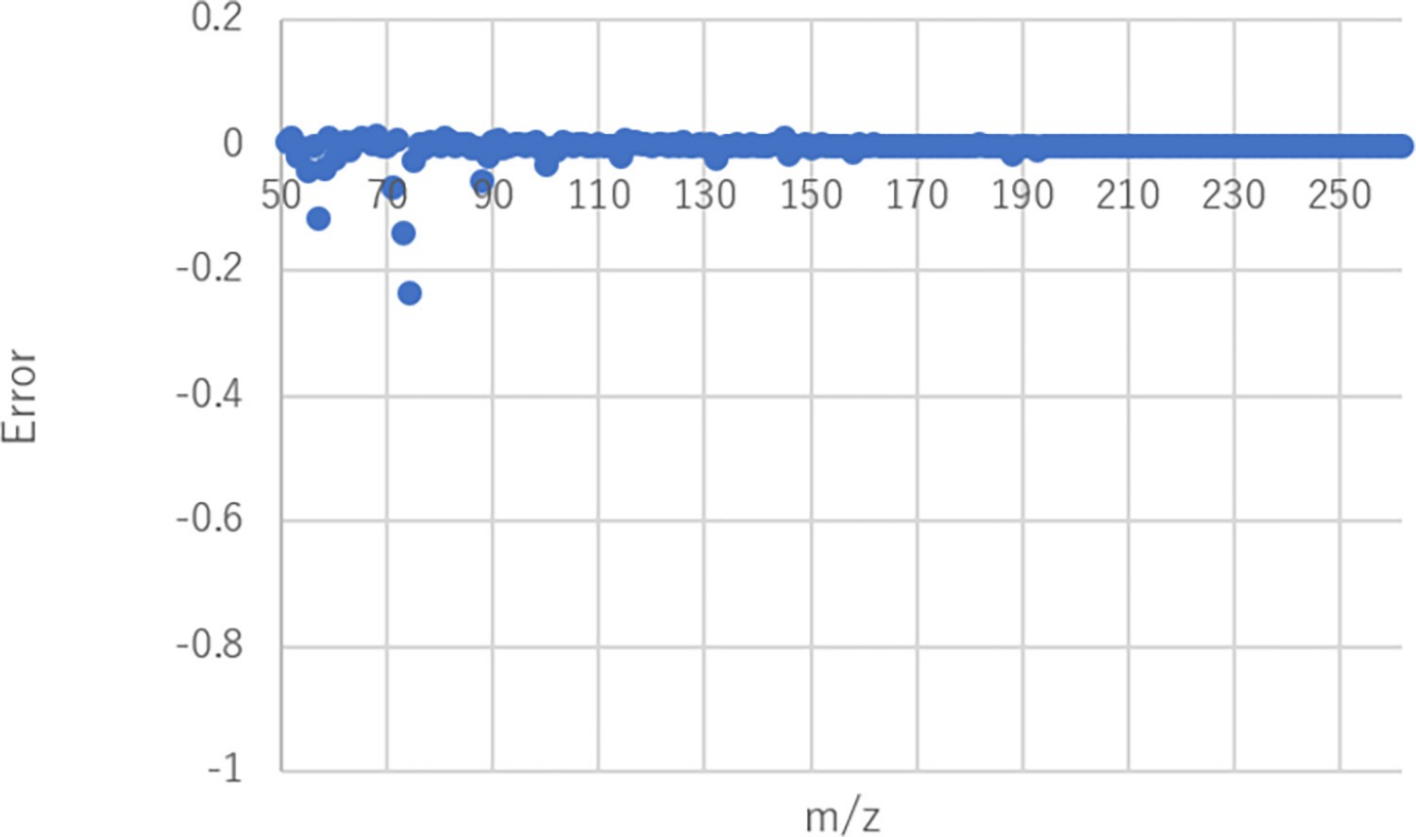
Error between obtained mass spectrum and approximated one using 2106 flavor molecules by nonnegative least square regression (‘fruit’).

**Fig 10:**
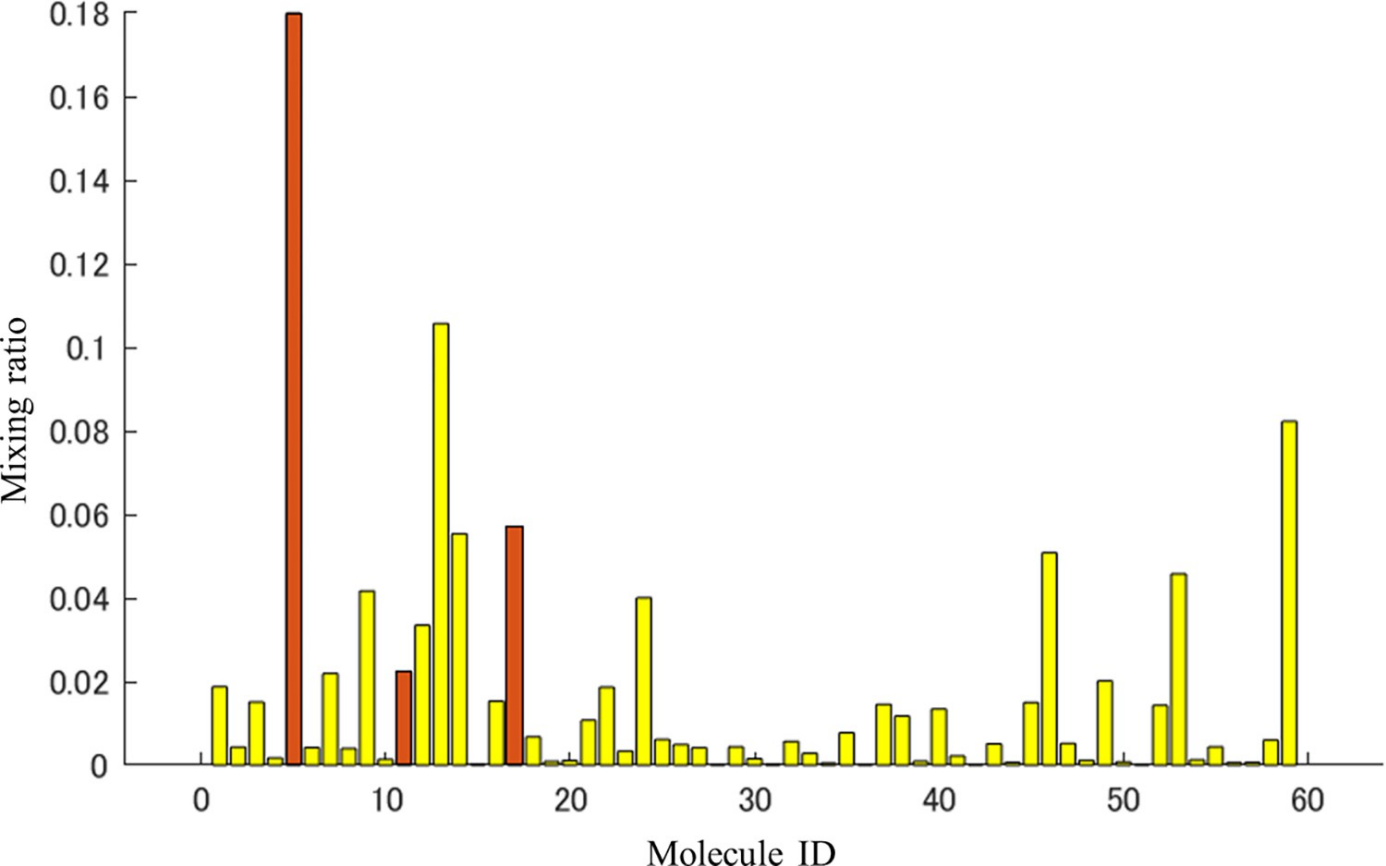
Mixing ratio of the molecules that contributed to the approximation in Fig 9. The molecules included in the original apple flavor are indicated by brown bars. Molecule names are shown in supplemental **S1 Table**.

Furthermore, we approximated the mass spectrum shown in **Fig 8** using the component mass spectra of the 2106 flavor molecules by the nonnegative least squares regression method. The MATLAB command (‘lsqnonneg’ with no option setting) was used to perform the nonnegative least squares regression method. The error between the approximated mass spectrum and the obtained mass spectrum in **Fig 8** is shown in **Fig 9** and the mixing ratio is shown in **Fig 10**. The approximation error of the mass spectrum was less than 0.00005 (sum of squares error) in the mass spectrum feature space obtained through the trained autoencoder. The number of molecules that contributed to the approximation was 59 out of 2106, which indicates that a highly accurate approximation was obtained with a relatively small number of types of molecule. Fifty-nine molecules are shown in supplemental **S1 Table**. The number of molecule types contained in the original apple flavor was three out of nine, and the mixing ratios were not low. Therefore, we can expect that the approximated mass spectrum might be slightly different from the original apple flavor, and its scent is similar to a fruit punch mixed with a non-apple fruit flavor. These findings should be verified by a sensory test.

**Fig 11:**
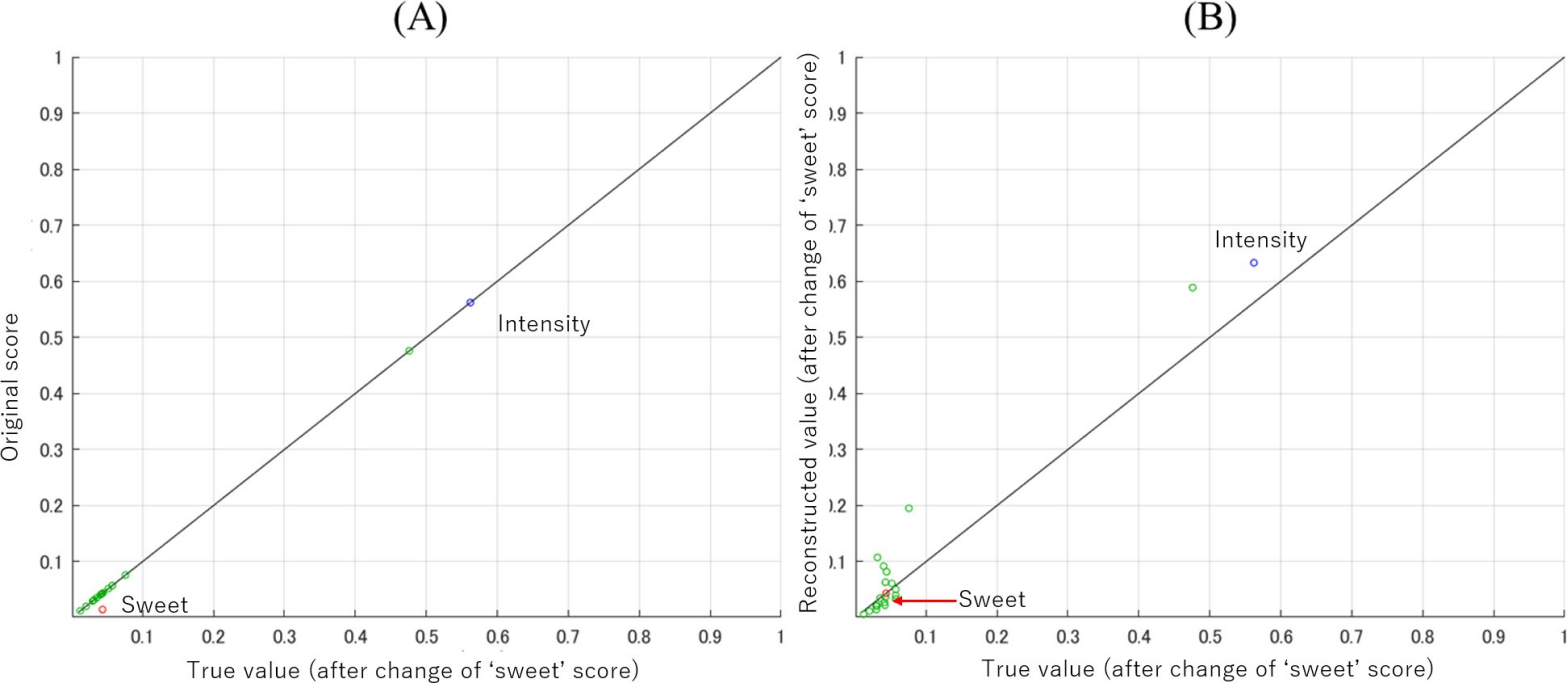
Predicted sensory test score **(A)**: The score for the original apple flavor (vertical axis) and the true value with the score of ‘sweet’ was multiplied by 3.0 (horizontal axis). **(B)**: The score for the explored mass spectral feature (vertical axis) and the true value with the score of ‘sweet’ was multiplied by 3.0 (horizontal axis). The blue, red and green plots are those of ‘intensity’, ‘sweet’ and others, respectively.

Then, we conducted another experiment on the mixed apple flavor. Here, we focused on the descriptor ‘sweet’ and conducted a series of similar experiments. The true value of ‘sweet’ was set to three times higher than the original one (**Fig 11(A)**), and the mass spectral feature was searched by the gradient descent method. The parameters of the search were *η*_*G*,*τ*_ = 0.1×0.999^*τ*^ and *m*_*i*_ = 1 for ‘sweet’ and *m*_*i*_ = 0 for other descriptors. The gradient descent was iterated 20,000 times. In reality, the error converged after about 200 iterations. **Fig 11(B)** shows the result in the sensory data space. As in the case of the descriptor ‘fruit’, the specified odor impression for ‘sweet’ was obtained since the red plot in the figure is on the diagonal line, and the other descriptors have different odor impressions from the original ones.

**Fig 12:**
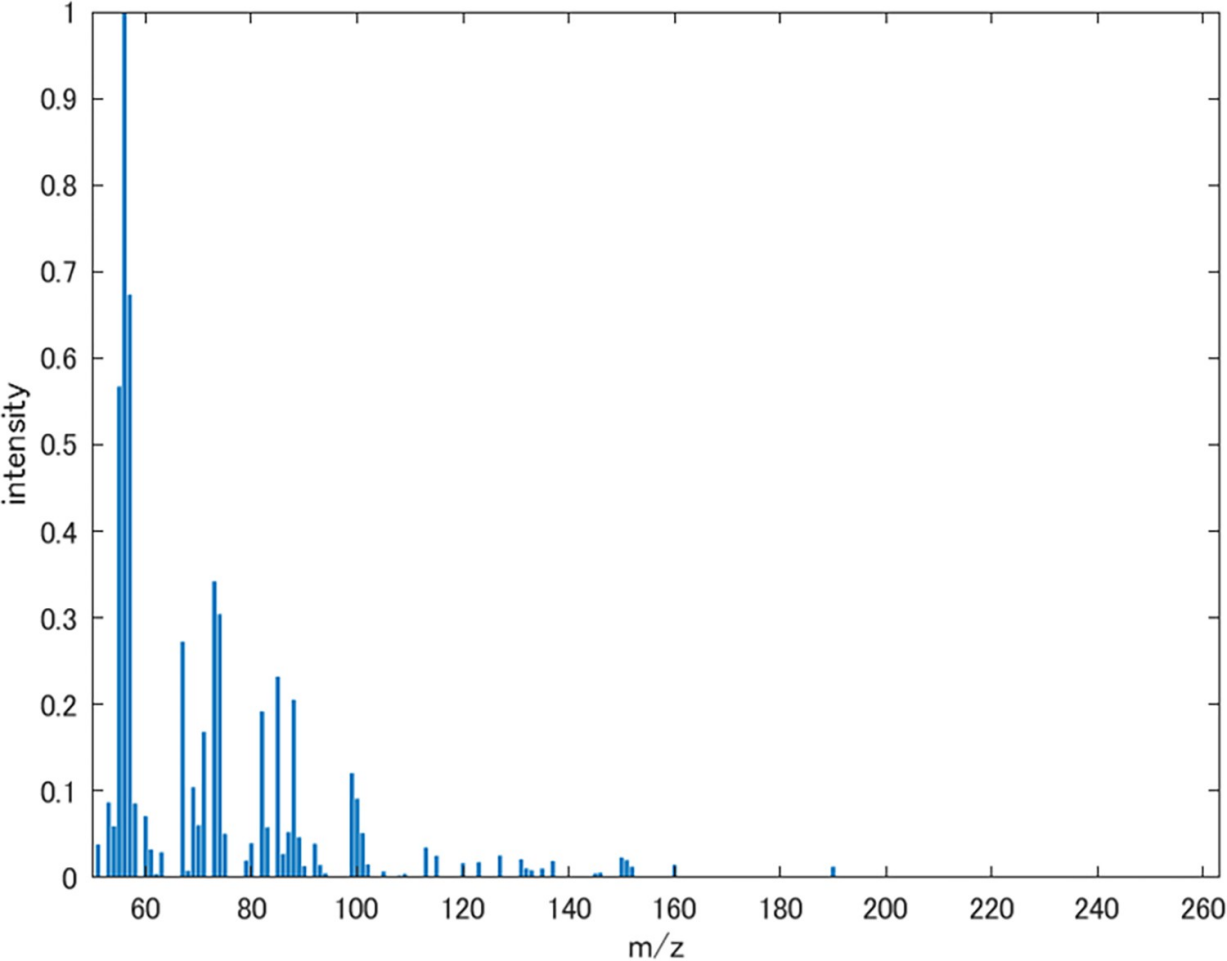
Mass spectrum obtained by applying Algorithm 1 in S1 Fig to the explored mass spectrum feature in the experiment for ‘sweet’.

Furthermore, using the same parameters, we conducted the mass spectrum search using **Algorithm 1 in S1 Fig** was conducted. The explored mass spectrum is shown in **Fig 12**. For the mass spectrum, the sum of squares of error on the mass spectrum feature space obtained through the autoencoder was 0.0165, which is comparable to that obtained for ‘fruit’. As in the case of the descriptor ‘fruit’, several peaks appeared in the high m/z region. However, compared with the original mass spectrum of the apple flavor, there was no significant change in the outline.

**Fig 13:**
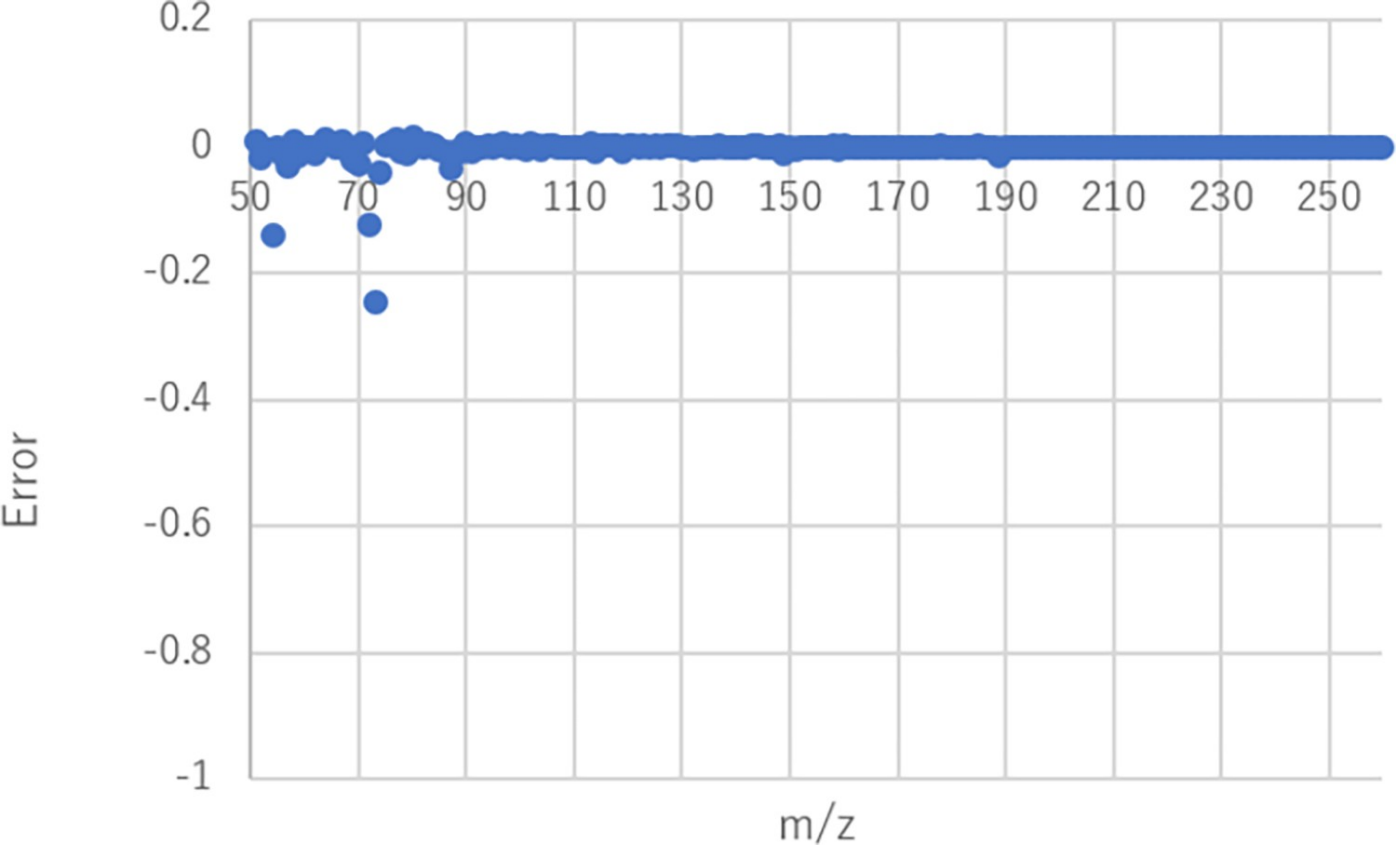
Error between obtained mass spectrum and approximated one using 2106 flavor molecules by nonnegative least square regression (‘sweet’).

**Fig 14:**
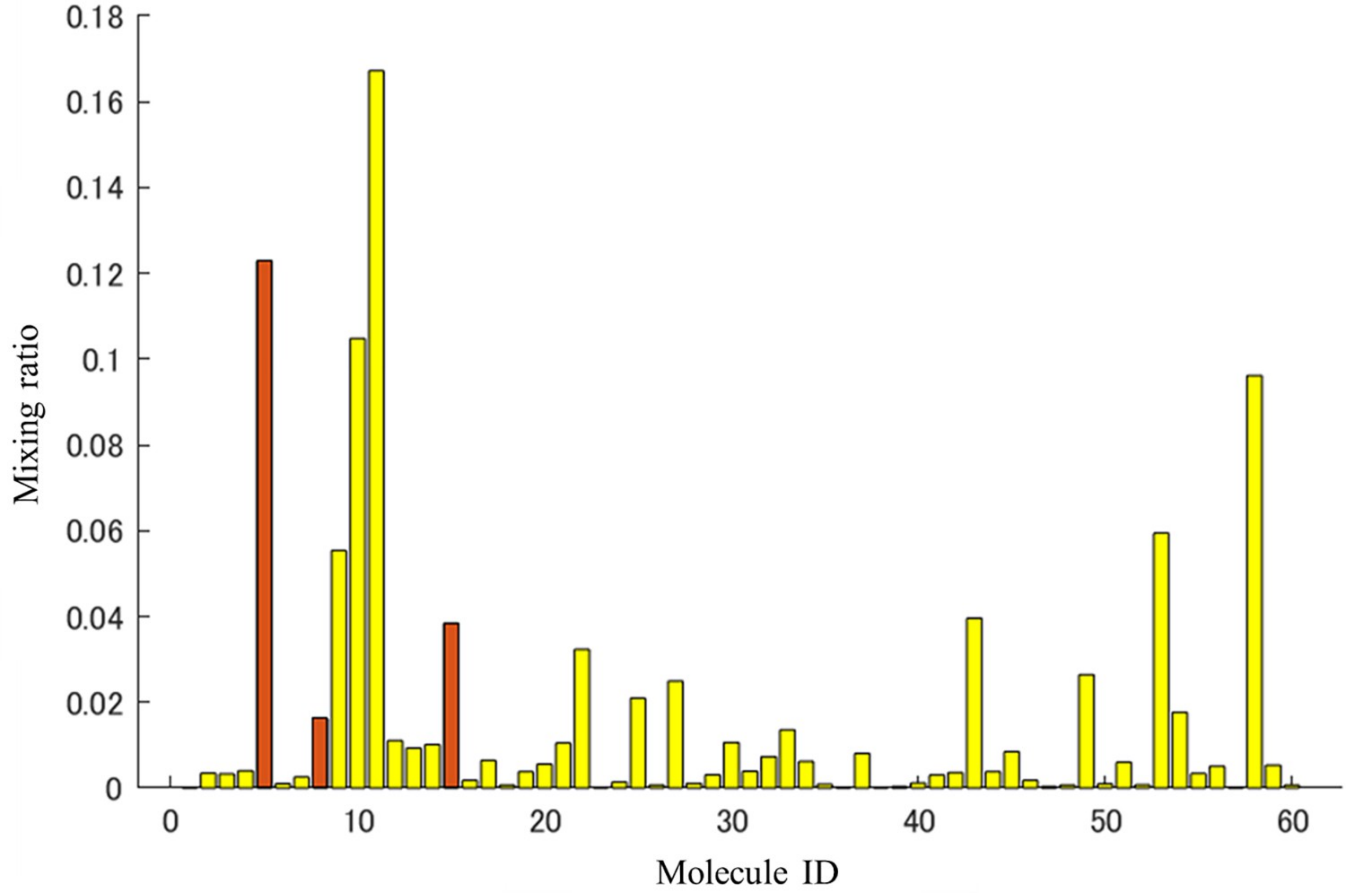
Mixing ratio of molecules that contributed to the approximation in Fig 13. The molecules included in the original apple flavor are indicated by brown bars. The list of the molecules is shown in **S2 Table**.

The mass spectrum shown in **Fig 12** was approximated using the 2106 molecules by solving the nonnegative least squares problem. The parameters of the calculation were the same as those for ‘fruit’. The error between the approximated mass spectrum and the obtained mass spectrum in **Fig 12** is shown in **Fig 13** and the mixing ratio is shown in **Fig 14**. The approximation error was less than 0.00005 (sum of squares error) in the mass spectrum feature space obtained through the trained autoencoder. The number of molecules that contributed to the approximation was 60 (supplemental **S2 Table**) out of 2106. The number of molecules contained in the original apple flavor was three out of nine, and the mixing ratios were not low. In this case, the odor mixture prepared by mixing 60 odorants is expected to smell more like the original apple flavor than in the case of the ‘fruit’ descriptor because the explored mass spectrum shown in **Fig 10** is similar to the original one, even though six of the nine molecules in the original flavor were absent. Note that the explored mass spectrum can contain noisy peaks. By limiting the types of molecule used to approximate the explored mass spectrum, it is possible to reduce such noises and to apply the desired bias. In other words, noises can be reduced by removing less-contributory molecules in a mixture ratio analysis, and the original outline of a flavor, e.g., apple flavor, can be maintained by approximating an explored mass spectrum without using other fruit-specific mass spectra.

## Conclusion

In this study, we proposed mathematical models and algorithms for calculating physicochemical features, i.e., the mass spectrum that realizes an intended odor impression. This is an attempt completely different from previous reports on predicting odor impression from the physicochemical structure parameters, which has not been so far reported except by us. From our previous report [9], this paper is extended in terms of odor mixtures and detailed algorithm descriptions.

The deep prediction model that made use of dimension reduction methods contributed to further improvement of the prediction accuracy. The experiment on a binary mixture (citral and vanillin) confirmed that this trained model can be used for odor mixtures as well as single molecules. Finally, it was found that the proposed method can express a mass spectrum corresponding to the change in odor impression for a complex odor mixture, and it was also possible to obtain the mixing ratio.

In this paper, we presented not only a new method for predicting the sensing data from the odor impression, but also a method for quantifying the mixing ratio of odorants to obtain the sensing data. For further improvement, the results presented in this paper need to be verified through sensory tests.

Since we can obtain the sensing data, i.e., mass spectrum, from odor impression, it is possible to make corresponding scent to have the identical sensing data. The odor approximation technique can extend generality of the proposed method [15]. The limitation of the model is that it is not always possible to realize an arbitrary odor impression, e.g., simultaneously warm and cold. It is necessary to understand the range of scents to be realized. Moreover, the uncertainty of the predicted sensing data and the corresponding mixing ratio will be investigated.

## Supporting information

S1 Fig Mass spectrum search from explored mass spectrum feature. (DOCX)

S1 Table Molecule IDs listed in Fig 10. (DOCX)

S2 Table Molecular IDs listed in Fig 14. (DOCX)
