# Supplementary material for: Exploration of sensing data to realize intended odor impression using mass spectrum of odor mixture
Source: PLoS One. 2022 Aug 17;17(8):e0273011. doi: 10.1371/journal.pone.0273011 (PMC9385042; doi:10.1371/journal.pone.0273011)
Supplement: S1 Fig — (DOCX) [file pone.0273011.s001.docx]

The algorithm is shown in **Algorithm 1**, **S1 Figure** using pseudo code. The function $MSE$ calculates and returns the MSE of the two arguments. The function $compare\_MSE$ calculates the MSE for each of the first three arguments with the fourth argument and returns the argument that minimizes the error and the error. The function $randi$ returns a random nonnegative integer whose maximum value is specified by the argument. The function $randperm$ a sequence obtained by randomly rearranging the numbers from 1 to the argument. The $\Delta$ is the update width that becomes smaller by multiplying the attenuation rate $\gamma$ with each iteration. The $flag_{\mathrm{updated}}$ is the indicator for incorporating randomness into the mass spectrum being searched for when the error stops decreasing during iteration, which prevents the search from getting stuck in local minima. The finally obtained $\boldsymbol{X}_{\mathrm{result}}^{\left( 1 \right)}$ is the mass spectrum corresponding to the explored mass spectrum feature.

**Algorithm 1**

| $\boldsymbol{X}^{\left( 1 \right)} = rand(size(\boldsymbol{X}^{\left( 1 \right)}))$  $\boldsymbol{X}^{(3)} = \boldsymbol{f}^{\left( 2,3 \right)}(\boldsymbol{f}^{\left( 1,2 \right)}\left( \boldsymbol{X}^{\left( 1 \right)} \right))$  $\boldsymbol{X}_{\mathrm{result}}^{\left( 1 \right)}=\boldsymbol{X}^{\left( 1 \right)}$  ${error}_{\mathrm{result}}=MSE(\boldsymbol{X}^{\left( 3 \right)},\boldsymbol{X}_{\mathrm{explored}}^{\left( 3 \right)})$  ${error}_{\min}={error}_{\mathrm{result}}$  $flag_{\mathrm{updated}}=0$  For $\tau=1:1:itr_{\max}$  $\boldsymbol{X}^{\left( 1 \right)}=\boldsymbol{X}_{\mathrm{result}}^{\left( 1 \right)}$  If $\left( flag_{\mathrm{updated}}==0 \right)$  $index_{\mathrm{random}}=randi\left( size\left( \boldsymbol{X}^{\left( 1 \right)} \right) \right)$  $\boldsymbol{X}^{\left( 1 \right)}\left( index_{\mathrm{random}} \right)=rand\left( \right)$  $\boldsymbol{X}^{\left( 1 \right)}=\boldsymbol{X}^{\left( 1 \right)}/\max\left( \boldsymbol{X}^{\left( 1 \right)} \right)$  End if  $\boldsymbol{X}_{+}^{\left( 1 \right)}=\boldsymbol{X}^{\left( 1 \right)}$  $\boldsymbol{X}_{-}^{\left( 1 \right)}=\boldsymbol{X}^{\left( 1 \right)}$  $\Delta=\Delta_{0}*\gamma^{\tau-1}$  $index_{\mathrm{randperm}}=randperm\left( size\left( \boldsymbol{X}^{\left( 1 \right)} \right) \right)$  For $index_{m/z}=1:1: size\left( \boldsymbol{X}^{\left( 1 \right)} \right)$  $index_{\mathrm{random}}=index_{\mathrm{randperm}}\left( index_{m/z} \right)$  $\boldsymbol{X}_{+}^{\left( 1 \right)}=\boldsymbol{X}_{\mathrm{result}}^{\left( 1 \right)}$  $\boldsymbol{X}_{\boldsymbol{-}}^{\left( 1 \right)}=\boldsymbol{X}_{\mathrm{result}}^{\left( 1 \right)}$  $\boldsymbol{X}_{+}^{\left( 1 \right)}\left( index_{\mathrm{random}} \right)=\boldsymbol{X}_{\boldsymbol{+}}^{\left( 1 \right)}\left( index_{\mathrm{random}} \right)+\Delta$  $\boldsymbol{X}_{-}^{\left( 1 \right)}\left( index_{\mathrm{random}} \right)=\boldsymbol{X}_{\boldsymbol{-}}^{\left( 1 \right)}\left( index_{\mathrm{random}} \right)-\Delta$  If $\left( \boldsymbol{X}_{\boldsymbol{-}}^{\left( 1 \right)}\left( index_{\mathrm{random}} \right)<0 \right)$  $\boldsymbol{X}_{\boldsymbol{-}}^{\left( 1 \right)}\left( index_{\mathrm{random}} \right)=0$  End if  $\boldsymbol{X}_{+}^{\left( 1 \right)}=\boldsymbol{X}_{+}^{\left( 1 \right)}/\max\left( \boldsymbol{X}_{+}^{\left( 1 \right)} \right)$  $\boldsymbol{X}_{\boldsymbol{-}}^{\left( 1 \right)}=\boldsymbol{X}_{\boldsymbol{-}}^{\left( 1 \right)}/\max\left( \boldsymbol{X}_{\boldsymbol{-}}^{\left( 1 \right)} \right)$  $\boldsymbol{X}^{(3)} = \boldsymbol{f}^{\left( 2,3 \right)}(\boldsymbol{f}^{\left( 1,2 \right)}\left( \boldsymbol{X}_{\mathrm{result}}^{\left( 1 \right)} \right))$  $\boldsymbol{X}_{\boldsymbol{+}}^{\left( 3 \right)} = \boldsymbol{f}^{\left( 2,3 \right)}(\boldsymbol{f}^{\left( 1,2 \right)}\left( \boldsymbol{X}_{+}^{\left( 1 \right)} \right))$  $\boldsymbol{X}_{\boldsymbol{-}}^{\left( 3 \right)} = \boldsymbol{f}^{\left( 2,3 \right)}(\boldsymbol{f}^{\left( 1,2 \right)}\left( \boldsymbol{X}_{-}^{\left( 1 \right)} \right))$  $\left[ \boldsymbol{X}_{\min}^{\left( 1 \right)},{error}_{\min} \right]=compare\_MSE\left( \boldsymbol{X}_{\mathrm{result}}^{\left( 1 \right)},\boldsymbol{X}_{+}^{\left( 3 \right)},\boldsymbol{X}_{-}^{\left( 3 \right)},\boldsymbol{X}_{\mathrm{explored}}^{\left( 3 \right)} \right)$  If $\left( {error}_{\mathrm{result}}>{error}_{\min} \right)$  ${error}_{\mathrm{result}}={error}_{\min}$  $\boldsymbol{X}_{\mathrm{result}}^{\left( 1 \right)}=\boldsymbol{X}_{\min}^{\left( 1 \right)}$  End if  End for  If $\left( \boldsymbol{X}_{\mathrm{result}}^{\left( 1 \right)}\neq\boldsymbol{X}^{\left( 1 \right)} \right)$  $flag_{\mathrm{updated}}=1$  Else  $flag_{\mathrm{updated}}=0$  End if  End for |
| --- |
