# Supplementary material for: Exploration of sensing data to realize intended odor impression using mass spectrum of odor mixture
Source: PLoS One. 2022 Aug 17;17(8):e0273011. doi: 10.1371/journal.pone.0273011 (PMC9385042; doi:10.1371/journal.pone.0273011)
Supplement: S1 Table — (DOCX) [file pone.0273011.s002.docx]

**S1 Table**: Molecule IDs listed in **Fig. 10**.

| ID | Molecular name | ID | Molecular name |
| --- | --- | --- | --- |
| 1 | Hexanedione; 2,3-Hexanedione; Acetyl butyryl; | 31 | Ethylacetophenone; 4-Ethylacetophenone; p-Ethylacetophenone |
| 2 | Anisaldehyde dimethyl acetal | 32 | Oxoglutaric acid; 2-Oxopentanedioic acid |
| 3 | Pyrazine | 33 | Paradol; |
| 4 | Butylpyridine; 3-Butylpyridine | 34 | Methylacetylpyrrole; 1-Methyl-2-acetylpyrrole; |
| 5 | Butyl alcohol; Butanol; 1-Butanol; n-Butanol; | 35 | Methanethiol isovalerate; S-Methyl 3-methylbutanethioate |
| 6 | Acetanisole; 4-Methoxyacetophenone; | 36 | Methyl beta-naphthyl ketone; Methyl 2-naphthyl ketone |
| 7 | Aspartic acid; L-Aspartic acid; | 37 | Acetic acid; Ethanoic acid; |
| 8 | Benzofurancarboxaldehyde; 2-Benzofurancarboxaldehyde; | 38 | Propenal; 2-Propenal; Prop-2-enal; Acrolien |
| 9 | Ethyl acrylate; | 39 | Methylfuranthiol; 2-Methyl-3-furanthiol; |
| 10 | Diisopropyl disulfide; Isopropyl disulfide; | 40 | Propanediol; 1,3-Propanediol; 1,3-Propylene glycol |
| 11 | Butyl isobutyrate; n-Butyl 2-methylpropanoate; | 41 | Naphthyl methyl ether; beta-Naphthyl methyl ether |
| 12 | Hexenyl formate; See cis-3-Hexenyl formate | 42 | Dimethyl tetrasulfide |
| 13 | Dimethyltetrahydrofuran; 2,5-Dimethyltetrahydrofuran | 43 | Octalactone; delta-Octalactone; 5-Octanolide; |
| 14 | Hexanedione; 3,4-Hexanedione; Dipropionyl; | 44 | Ethyl levulinate propylene glycol ketal |
| 15 | Elemicin | 45 | Allyl hexanoate; Allyl caproate; |
| 16 | Pyrrole; | 46 | Hexenyl octanoate; cis-3-Hexenyl octanoate |
| 17 | Isobutyric acid; 2-Methylpropanoic acid; | 47 | Methylformylpyrrole; N-Methyl-2-formylpyrrole |
| 18 | Propylphenethyl alcohol; alpha-Propylphenethyl alcohol; | 48 | Dimethylquinoxaline; 2,3-Dimethylquinoxaline |
| 19 | Diethylmethylpyrazine; 2,3-Diethyl-5-methylpyrazine; | 49 | Ethyl acetylpropionate; Ethyl 2-acetylpropionate |
| 20 | Dimethyl sulfoxide; DMSO; Methylsulfinylmethane; | 50 | Acetoxybutyl acetate; 4-Acetoxybutyl acetate |
| 21 | Methyl octanethioate; S-Methyl octanethioate | 51 | Butyl anthranilate; |
| 22 | Methyl geranate; Methyl 3,7-dimethyl-trans-2,6-octadienoate | 52 | Methylthioethanol; 2-(Methylthio)ethanol; |
| 23 | Ethyl tiglate; Ethyl trans-2-methylbut-2-enoate; | 53 | Pentanedione; 2,3-Pentanedione; Acetyl propionyl; |
| 24 | Ethylbutyric acid; 2-Ethylbutyric acid; 2-Ethylbutanoic acid; | 54 | Hydroxybutanoic acid lactone; gamma-Butyrolactone |
| 25 | Diethyl maleate | 55 | Methylbenzofuran; 2-Methylbenzofuran |
| 26 | Methyl acetylanthranilate; Methyl N-acetylanthranilate | 56 | Diallyl tetrasulfide |
| 27 | Mercaptomethyllpentanone; 2-Mercapto-2-methylpentan-4-one | 57 | Cyclohexyl benzoate |
| 28 | Methyl dimethylanthranilate; Methyl N,N-dimethylanthranilate | 58 | Acetyldimethylfuran; 2-Acetyl-3,5-dimethylfuran |
| 29 | Ethyl methoxyacetate; ethyl 2-methoxyacetate | 59 | Acetol; 1-Hydroxypropan-2-one |
| 30 | Methylcoumarin; 6-Methylcoumarin; | - | - |
