# Supplementary material for: Exploration of sensing data to realize intended odor impression using mass spectrum of odor mixture
Source: PLoS One. 2022 Aug 17;17(8):e0273011. doi: 10.1371/journal.pone.0273011 (PMC9385042; doi:10.1371/journal.pone.0273011)
Supplement: S2 Table — (DOCX) [file pone.0273011.s003.docx]

**S2 Table**: Molecule IDs listed in **Fig. 14**.

| ID | Molecular name | 番号 | Molecular name |
| --- | --- | --- | --- |
| 1 | Anisaldehyde dimethyl acetal | 31 | Methoxymethylpropane; 2-Methoxy-2-methylpropane; MTBE |
| 2 | Mercaptopropanone; 1-Mercapto-2-propanone | 32 | Oxoglutaric acid; 2-Oxopentanedioic acid |
| 3 | Pyrazine | 33 | Methyltetrahydrofuran; 2-Methyltetrahydrofuran |
| 4 | Butylpyridine; 3-Butylpyridine | 34 | Paradol; |
| 5 | Butyl alcohol; Butanol; 1-Butanol; n-Butanol; | 35 | Methylacetylpyrrole; 1-Methyl-2-acetylpyrrole; |
| 6 | Aspartic acid; L-Aspartic acid; | 36 | Methyl beta-naphthyl ketone; Methyl 2-naphthyl ketone |
| 7 | Benzofurancarboxaldehyde; 2-Benzofurancarboxaldehyde; | 37 | Acetic acid; Ethanoic acid; |
| 8 | Butyl isobutyrate; n-Butyl 2-methylpropanoate; | 38 | Methylnaphthalene; 1-Methylnaphthalene; |
| 9 | Hexenyl formate; See cis-3-Hexenyl formate | 39 | Methyl vanillate; Methyl 4-hydroxy-3-methoxybenzoate; |
| 10 | Dimethyltetrahydrofuran; 2,5-Dimethyltetrahydrofuran | 40 | Methylpyrazine; 2-Methylpyrazine; |
| 11 | Dimethylhexane; 2,2-Dimethylhexane | 41 | Isobutyl tiglate; Isobutyl 2-methylcrotonate |
| 12 | Ethyl heptanoate; | 42 | Allyl hexanoate; Allyl caproate; |
| 13 | Pyrrole; | 43 | Hexenyl octanoate; cis-3-Hexenyl octanoate |
| 14 | Methylpentanal; 2-Methylpentanal; | 44 | Ethyl acetylpropionate; Ethyl 2-acetylpropionate |
| 15 | Isobutyric acid; 2-Methylpropanoic acid; | 45 | Glycine; Aminoacetic acid; |
| 16 | Methyl thioisobutyrate; S-Methyl isothiobutyrate | 46 | Methylthioethanol; 2-(Methylthio)ethanol; |
| 17 | Propylphenethyl alcohol; alpha-Propylphenethyl alcohol; | 47 | Hydroxybutanoic acid lactone; gamma-Butyrolactone |
| 18 | Diethylmethylpyrazine; 2,3-Diethyl-5-methylpyrazine; | 48 | Methylbenzofuran; 2-Methylbenzofuran |
| 19 | Dimethyl sulfoxide; DMSO; Methylsulfinylmethane; | 49 | Acetaldehyde ethyl cis-3-hexenyl acetal ; Leaf acetal |
| 20 | Methyl octanethioate; S-Methyl octanethioate | 50 | Cyclohexyl benzoate |
| 21 | Amyl methyl disulfide; Methyl pentyl disulfide; 2,3-Dithiaoctane | 51 | Ethyl mercaptoacetate; Ethyl 2-mercaptoacetate |
| 22 | Ethylbutyric acid; 2-Ethylbutyric acid; 2-Ethylbutanoic acid; | 52 | Acetyldimethylfuran; 2-Acetyl-3,5-dimethylfuran |
| 23 | Divanillin; | 53 | Pentanol; 2-Pentanol; |
| 24 | Diethyl maleate | 54 | Ethyl methylpentanoate; Ethyl 3-methylpentanoate; |
| 25 | Ethyl butenoate; Ethyl 2-Butenoate; Ethyl crotonate; | 55 | Ionone epoxide; beta-Ionone epoxide; 5,6-epoxy-beta-ionone |
| 26 | Potassium acetate; | 56 | Hydroxymethoxybenzaldehyde; 2-Hydroxy-4-methoxybenzaldehyde |
| 27 | Angelic acid; cis-2-Methylbut-2-enoic acid | 57 | Ethyl vanillate; Ethyl 4-hydroxy-3-methoxybenzoate; |
| 28 | Naphthalenethiol; 2-Naphthalenethiol; | 58 | Acetol; 1-Hydroxypropan-2-one |
| 29 | Methylcoumarin; 6-Methylcoumarin; | 59 | Diethyl malonate; Diethyl propane-1,3-dioate; |
| 30 | Isobutyl heptanoate; 2-Methylpropyl heptanoate; | 60 | Methylpropionylfuran; 2-Methyl-5-propionylfuran |
